# Lung Adenocarcinoma Originates from Retrovirus Infection of Proliferating Type 2 Pneumocytes during Pulmonary Post-Natal Development or Tissue Repair

**DOI:** 10.1371/journal.ppat.1002014

**Published:** 2011-03-31

**Authors:** Claudio Murgia, Marco Caporale, Ousman Ceesay, Gabriella Di Francesco, Nicola Ferri, Vincenzo Varasano, Marcelo de las Heras, Massimo Palmarini

**Affiliations:** 1 Medical Research Council – University of Glasgow Centre for Virus Research, Institute of Infection, Inflammation and Immunity, College of Medical, Veterinary and Life Sciences, University of Glasgow, United Kingdom; 2 Istituto G. Caporale, Teramo, Italy; 3 Dipartimento di Scienze Cliniche Veterinarie, Facolta' di Medicina Veterinaria, Universita' di Teramo, Italy; 4 Facultad de Veterinaria, Universidad de Zaragoza, Zaragoza, Spain; Fred Hutchinson Cancer Research Center, United States of America

## Abstract

Jaagsiekte sheep retrovirus (JSRV) is a unique oncogenic virus with distinctive biological properties. JSRV is the only virus causing a naturally occurring lung cancer (ovine pulmonary adenocarcinoma, OPA) and possessing a major structural protein that functions as a dominant oncoprotein. Lung cancer is the major cause of death among cancer patients. OPA can be an extremely useful animal model in order to identify the cells originating lung adenocarcinoma and to study the early events of pulmonary carcinogenesis. In this study, we demonstrated that lung adenocarcinoma in sheep originates from infection and transformation of proliferating type 2 pneumocytes (termed here lung alveolar proliferating cells, LAPCs). We excluded that OPA originates from a bronchioalveolar stem cell, or from mature post-mitotic type 2 pneumocytes or from either proliferating or non-proliferating Clara cells. We show that young animals possess abundant LAPCs and are highly susceptible to JSRV infection and transformation. On the contrary, healthy adult sheep, which are normally resistant to experimental OPA induction, exhibit a relatively low number of LAPCs and are resistant to JSRV infection of the respiratory epithelium. Importantly, induction of lung injury increased dramatically the number of LAPCs in adult sheep and rendered these animals fully susceptible to JSRV infection and transformation. Furthermore, we show that JSRV preferentially infects actively dividing cell *in vitro*. Overall, our study provides unique insights into pulmonary biology and carcinogenesis and suggests that JSRV and its host have reached an evolutionary equilibrium in which productive infection (and transformation) can occur only in cells that are scarce for most of the lifespan of the sheep. Our data also indicate that, at least in this model, inflammation can predispose to retroviral infection and cancer.

## Author Summary

The identification of cells that give origin to cancer is critical in order to design effective therapeutic strategies. To this end, the early stages of cancer are the most informative but they are seldom associated with clinical symptoms and therefore pass unnoticed in human patients. Studies on animal tumors are invaluable to this research area. In this study, we determined the cells originating an infectious lung cancer of sheep (ovine pulmonary adenocarcinoma, OPA) that is similar to some forms of human pulmonary adenocarcinoma. OPA is caused by a virus known as Jaagsiekte sheep retrovirus (JSRV). We show that OPA is caused by JSRV infection of proliferating type 2 pneumocytes (lung alveolar proliferating cells, LAPCs). We show that young animals possess abundant LAPCs and are highly susceptible to JSRV infection while healthy adult sheep exhibit a relatively low number of LAPCs and are resistant to OPA induction. However, adult sheep were susceptible to JSRV infection when the presence of LAPCs was stimulated by induction of a mild injury to the respiratory epithelium. Thus, our study identifies the cells originating lung adenocarcinoma in OPA and shows that inflammation to the respiratory epithelium can predispose to retrovirus infection and cancer.

## Introduction

Retroviruses have been instrumental in understanding the genetic basis and the fundamental molecular mechanisms leading to cancer [1]. Studies on the pathogenesis of retrovirus induced malignancies have also contributed to our understanding of the cells that give origin to cancer and the role played by stem and progenitor cells in these processes [2]. The “cancer stem cell” (CSC) hypothesis postulates that cancer is initiated and sustained by adult stem cells [3]–[4]. A growing body of experimental evidence is supporting the presence of CSCs in haematological malignancies and in some solid tumours. However, the presence and significance of CSCs is object of considerable debate particularly in slow turnover organs such as the lungs [5]–[7]. Identifying the cells that give origin to cancer is critical both to understand the basic carcinogenetic processes but also to devise appropriate therapeutic strategies.

Most retroviruses induce transformation of hematopoietic cells but there are a few notable exceptions causing sarcomas, nephroblastomas, mammary carcinomas, nasal and lung adenocarcinomas in a variety of animal species [8]. Ovine pulmonary adenocarcinoma (OPA) is a naturally occurring (and experimentally inducible) lung cancer of sheep caused by a retrovirus known as Jaagsiekte sheep retrovirus (JSRV) [9]–[11]. OPA is a common disease of sheep in most geographical areas of the world. Interestingly, the disease shares several clinical and histological features with some forms of human lung adenocarcinomas. Therefore, OPA represents an excellent animal model with great potential to contribute significantly to our understanding of retroviral pathogenesis, lung tumorigenesis and pulmonary biology [9], [12]–[13].

JSRV is the only oncogenic virus that causes a naturally occurring lung adenocarcinoma. Interestingly, in contrast to the overwhelming majority of oncogenic retroviruses, JSRV is a replication-competent virus that possesses a structural protein (the viral envelope, Env) that acts as a dominant oncoprotein [14]–[16]. Expression of the JSRV Env is sufficient to induce cell transformation *in vitro* in a variety of cell lines [13]–[15], [17]–[22] and importantly *in vivo* in both experimental mice models and in lambs [23]–[24]. Thus, productive virus infection and cell transformation are mutually dependent in OPA and this creates an “evolutionary dilemma” as, at face value, abundant viral replication is entirely dependent on tumor development in the host. The JSRV Env is believed to induce cell transformation via the activation of a variety of signal transduction pathways including the PI-3K/Akt and Ras-MEK-MAPK [13], [20], [22],[25]–[27].

Experimentally, intratracheal inoculation of concentrated JSRV viral particles in young lambs induces OPA in the overwhelming majority of animals with a very short incubation period (varying from a few weeks to a few months) [28]–[29]. There is a clear age-dependent susceptibility to experimentally induced OPA in lambs while it is not possible (or extremely difficult) to reproduce the disease in adult sheep [29]. These data suggest that there is a different availability of the target cells of JSRV transformation in animals of a different age. The age-susceptibility to OPA induction does not appear to be related to expression of the receptor in target cells or to a differential immune response towards the virus. Indeed, the cellular receptor for the virus (Hyaluronidase-2, Hyal-2) is ubiquitously expressed [16], [29] and this virus can infect several cell types *in vitro* and *in vivo* [30]–[33]. In addition, JSRV naturally or experimentally-infected animals do not mount a significant immune response, likely as a result of tolerance induced by expression of JSRV-related endogenous retroviruses (enJSRVs) which are present in the genome of all domestic and wild sheep [34]–[37].

In OPA affected sheep, abundant expression of JSRV proteins are confined to the tumor cells although viral RNA and DNA can be detected by sensitive PCR assays in a variety of cells of the lymphoreticular system [30]–[31], [38]. In sheep naturally infected with JSRV and with no neoplastic lesions, JSRV can be detected only in lymphoid tissues [39]. OPA tumours, similar to some human lung adenocarcinomas, are formed by secretory cells of the distal pulmonary tract; predominantly alveolar type 2 pneumocytes and less commonly the non-ciliated bronchial cells of the terminal bronchioli (Clara cells; see note at the end of the text on the usage of this term) [40]–[42]. Interestingly, a putative bronchioalveolar stem cell (BASC) has been identified in mice lungs although its presence in other species, including humans, has not been established with certainty [43]. It has been proposed that BASCs have the capacity to originate both Clara cells and type 2 pneumocytes and to be the cell origin of lung adenocarcinoma in mice in response to oncogenic K-ras [43]. However the significance of BASCs in physiological and pathological processes and the origin of lung adenocarcinoma are under debate [44]–[45].

In order to identify the target cells of JSRV infection and transformation we performed a series of *in vivo* studies in experimentally infected lambs and adult sheep. Furthermore, we derived a JSRV-based vector in order to assess the ability of this virus to infect non-dividing cells *in vitro*. In this study we identified the cells target of JSRV infection and transformation and provide important insights into lung biology, pulmonary carcinogenesis and retroviral pathogenesis.

## Methods

### Ethics statement

All experimental procedures carried out in this study are included in Project Licence 60/3905 approved by the Home Office of the United Kingdom in accordance to the “animals (scientific procedures) act 1986”. Experiments carried out at the Istituto G. Caporale were also detailed in protocol number 3315 approved by the Italian Ministry of Health (Ministero della Salute) in accordance with Council Directive 86/609/EEC of the European Union.

### Virus stock preparation

Viral stocks used in all these experiments were produced in rat 208F.JSRV_21_ cells as already described [46]. Briefly, 208F.JSRV_21_ derive from 208F cells [47] stably transfected with a plasmid expressing the JSRV_21_ infectious molecular clone [11]. 208F.JSRV_21_ cells were plated at 80% confluence and supernatants were collected after 24, 48 and 72 h. Virus was concentrated by ultracentrifugation [300×] as previously described [11] and resuspended in 1×TNE buffer (100mM NaCl, 10 mM Tris, 1 mM EDTA). The infectious titer for JSRV cannot be easily calculated *in vitro*, because of the lack of a convenient tissue culture system for this virus. In order to infect animals with the same amount of JSRV, pellets from various virus preparations were pooled into a single stock, divided into 1 ml aliquots and stored at −80°C until use. In all the experiments described below, each animal received the same amount of virus stock. In a related study, the same JSRV preparation used here, induced OPA in 4 of 4 experimentally infected lambs within 5 months after inoculation (Caporale and Palmarini, unpublished).

### *In vivo* studies

Animal studies were performed at the Istituto G. Caporale (Teramo, Italy) and at the University of Glasgow. Prior to experimental infections all animals were anaesthetised with sodium pentobarbital anesthesia, and all efforts were made to minimize suffering. To facilitate the detection of infected cells, JSRV (1 ml) was inoculated directly into the accessory bronchus of the cranial lobe of the right lung by fiber-optic bronchoscopy. Sheep used in this study were females between 3 and 5 year old of either bergamasca cross-breed (study 1, 2 & 4) or blackface breed (study 3) unless otherwise indicated. Three independent studies were performed as follow.

#### Study 1: Age related susceptibility to JSRV infection

Four 2-day old lambs and 4 adult sheep were anesthetized and inoculated with JSRV, as described above. Two animals were used as mock inoculated controls. Ten days post infection animals were euthanized, the lungs removed from the thoracic cavity and examined for the presence of macroscopic lesions. Samples from respectively 8 (in lambs) and 16 (in sheep) regions from the cranial lobe were collected and fixed overnight in 10% buffered formalin and embedded in paraffin. Tissue sections were examined by immunohistochemistry and immunofluorescence as described below.

#### Study 2: Bronchioalveolar proliferation in lambs and adult sheep

Lung tissues were collected at post-mortem from adult sheep (n = 2) and 4 lambs (2–4 day old). Tissues were collected from 4 different lobes of the lungs and fixed overnight in 10% buffered formalin. Tissues were examined for bronchiolar alveolar cell proliferation from ten sections from each animal as described below.

#### Study 3: Induction of mild lung injury in adult sheep

Mild lung injury was induced in adult sheep using 3- methylindole (3MI, Sigma). Four adult sheep were divided in two groups. All animals were weighted and fasted 12 hours before dosing. Group 1 received 0.25 g/kg body weight of 3MI (Sigma) dissolved in 50 ml of corn oil (Sigma) and administrated using a stomach tube attached to a syringe. Group II served as control and received a similar amount of corn oil. After 48 hours all animals were euthanized and lung tissue were collected for histological and immunofluorescence analysis to assess the injury and cell proliferation.

#### Study 4: Infection of adult sheep with or without lung injury

Ten adult sheep were divided in two groups of 5 animals each. Group 1 received 0.25 g 3MI/kg body wt as described above. Group 2 served as control and received a similar amount of corn oil. After 48 h all animals from group 1 and 2 were infected with JSRV as described above and euthanized 10 days post-infection. Samples of lung tissues were collected at post-mortem and processed as above.

### Naturally occurring OPA tumour samples

Formalin-fixed, paraffin-embedded OPA tumour samples from naturally occurring (n = 6) and experimentally induced (n = 2) cases were obtained from the Department of Veterinary Pathology, University of Zaragoza. All tumour samples were previously diagnosed as JSRV positive by immunohistochemistry as already described [23], [38], [48]. Four serial sections for each tumour were analysed by immunofluorescence as described below.

### Immunofluorescence and immunohistochemistry

Tissue sections were deparaffinised and hydrated using standard procedures. Antigen retrieval was performed using citrate buffer (pH6) and pressure cooker heating. To quench endogenous peroxidase, sections were incubated in 3% H_2_O_2_ diluted in methanol or PBS for 30 minutes. Sections were incubated overnight at 4°C with the following primary antibodies: polyclonal rabbit anti pro-SP-C (Seven Hills Bioregagents or Chemicon, dilution 1∶4000), monoclonal mouse anti Ki67 (DAKO, 1∶2000), mouse monoclonal anti JSRV Env (1∶200, kindly provided by Dusty Miller) [24], [49]. For CC10 detection we used either a polyclonal rabbit (Proteintech) or mouse (Dundeecell products) antisera generated against full length recombinant bovine CC10. Mouse CC10 was detected using goat anti-mouse CC10 clone T18 (Santa Cruz; 1∶200). Immunofluorescence detection was performed using the following labelled secondary antibodies: goat anti-mouse Alexa488, donkey anti-rabbit Alexa-555, donkey anti-rabbit Alexa 488. SP-C was detected using horseradish peroxidase (HRP)-conjugated donkey anti-rabbit secondary antibody (1∶6000) by tyramide signal amplification (TSA; Perkin-Elmer Life Science Products) while Ki67 was detected using donkey anti-mouse Alexa488 or Alexa-555. Slides were mounted with medium containing DAPI (Vectashield; Vector Laboratories). Immunohistochemistry was performed with Dako supervision system (DAKO) and slides were counterstained with haematoxylin. Confocal images were analysed and merged using Image-pro analyser 7 software (MediaCybernetics). Histological images were captured using cell∧D software (Olympus). Proliferation analysis was performed by counting SP-C/Ki67 double positive cells in the entire 10 lung sections for each animal using a Leica TCS SP2 confocal microscope. Numbers of double positive cells were normalized to the sectioned area using Image-pro analyser 7 Software. Bronchiolar cell proliferation was determined by counting the number of CC10^+^/Ki67^+^ cells in 100 terminal bronchioli for each animal.

### Plasmids

The JSRV-based vector employed in this study was derived from the JSRV_21_ infectious molecular clone pCMV2JS21 [11] and was termed pCJS-EfGFP-mC. Most of the JSRV *gag* and *pol* have been deleted and replaced by a cassette containing the promoter of the human elongation factor 1 α (EF1α) driving the enhanced green fluorescent protein (eGFP). The EF1α-eGFP cassette was derived from pDRIVE5-GFP-3 (InvivoGen). In addition, pCJS-EfGFP-mC also contains the woodchuck hepatitis post-transcriptional regulatory element (WPRE; before the *env* splice acceptor) [50]–[51] derived from pCCLcPPTPGKEGFPLTRH1shSOD1 (Addgene Inc.). In pCJS-EfGFP-mC, the JSRV *env* has also been deleted and replaced with the cDNA expressing the mCherry fluorescent protein, followed by two copies of the Mason-Pfizer constitutive transport element (CTE) [52]–[53]. The packaging plasmid pCMVGPP-MX-4CTE expresses the JSRV Gag, Pro and Pol genes and derives from plasmid pGPP-MX by the addition of 3 additional CTE copies. pGPP-MX has been already described [23]. pCMV-SX2.JS-env expresses the JSRV Env under the control of the CMV immediate early promoter and was derived from the pSX2.Jenv (a gift by Dusty Miller) [33], [54]. pCDNA3-HA-Sam68 is an expression plasmid for the RNA binding protein Sam68 and was a gift from David Shalloway [55]. Plasmids pCSGW-GFP (HIV-based vector), p8.2 and pMD.G have been described previously [56].

### Cells

293T cells and sheep choroid plexus (SCP) cells were grown in Dulbecco's modified Eagle's medium and Iscove's modified Dulbecco's medium respectively supplemented with 10% fetal bovine serum at 37°C, with 5% CO_2_ and 95% humidity.

### JSRV vector production

Particles of a JSRV-based viral vector (JS-EeGFP-mCherry) were produced by co-transfecting 293T cells with pCJS-EfGFP-mC, pCMVGPP-MX-4CTE, pCMV-SX2.JS-env and pCDNA3-HA-Sam68 plasmids essentially as described previously [23]. Viral particles were collected from supernatants of transfected cells, 24 and 48 h post-transfection, filtered through 0.45 µm filters (Millipore) and concentrated [200×] by ultracentrifugation as described previously [23]. A lentiviral vector (HIV-GFP) was used as control and prepared exactly as above by co-transfecting 293T cells with pCSGW-GFP, p8.2 and pMD.G.

### *In vitro* vector transduction

Target cells synchronization was established by culturing SCP cells in the presence of 0.2% fetal bovine serum (FBS) for 72 h. Synchronized SCP cells were then seeded at 5×10^4^ cells/well in 6 well plates and treated for 25 h with 5 µg aphidicolin (Sigma). Target cells were infected with serial dilutions of the JSRV or HIV-based vector in presence of polybrene [57], [58]. Transduction controls included infection with heat-treated vector preparations (65°C/30′). 12 h post-infection, cells were washed three times with phosphate-buffered saline and incubated for further 48 h in the presence or absence of aphidicolin. Viral titers were expressed as fluorescence forming foci/ml and were determined by counting foci of GFP positive cells 48 h post-infection. Cellular DNA content was determined by staining cells with 7-Aminoactinomycin D (7AAD, Invitrogen) and measuring fluorescence in a Beckman Coulter flow cytometer. SCP cells were harvested by trypsinization and incubated for 1 h with 25 µg/ml 7AAD, 0.03% saponin (Sigma) and 1% BSA (Sigma). Cells were then transferred in 500 µl of 1×PBS and the proportion of cells in G0/G1, S and G2/M phases was estimated using expo32 software (Beckman Coulter) and counting 20000 events.

## Results

### Phenotype of naturally occurring OPA tumours

Ultrastructural, histological and immunophenotyping studies have shown that OPA tumours, similarly to some forms of human adenocarcinomas, are formed by type 2 pneumocytes and to a lesser extent by Clara cells [40], [42], [59]–[62]. No data are available in the literature on whether JSRV is expressed in both these cell types in the OPA tumours.

**Figure 1:**
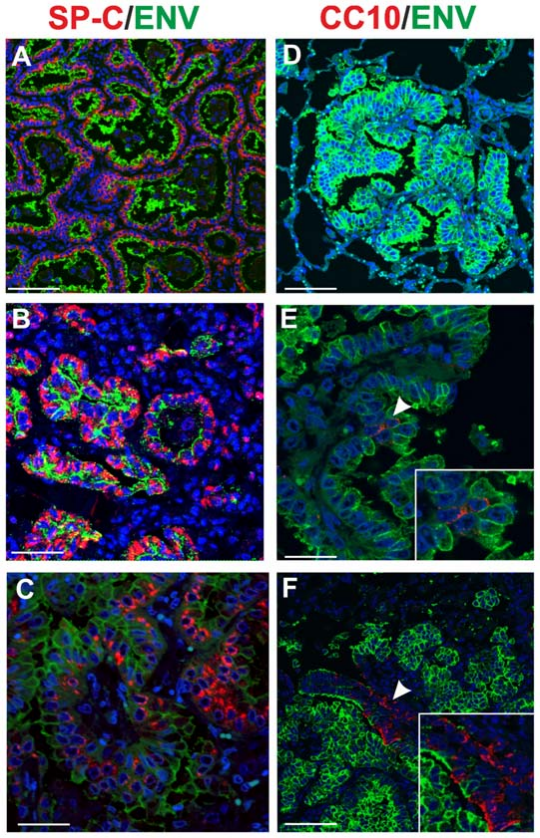
Phenotype of tumor cells in naturally occurring OPA. Immunofluorescence of lung tumor sections from sheep affected by OPA. Panel A–C show tumor sections analyzed by confocal microscopy using antibodies towards SP-C (showed in red), the JSRV Env (showed in green) and the appropriate secondary antibodies as described in Materials and Methods. Panels D–F show tumor sections analyzed by confocal microscopy using antibodies towards CC10 (showed in red), and the JSRV Env (showed in green). Nuclei were stained with DAPI and are shown in blue. Arrows in panel E indicates a CC10+ cells where JSRV Env expression is not detectable. The insert in panel E shows a larger magnification of the area indicated by the arrows. Scale bars: A,F = 75 µm, B = 43 µm, C = 34 µm, D = 89 µm, E = 29 µm.

Here, we analysed by immunofluorescence and confocal microscopy serial tumor sections collected from six sheep with late stages of naturally occurring OPA and two lambs with experimentally induced disease, in order to characterize both the phenotype of the cells forming the neoplasm and viral expression. Type 2 pneumocytes and Clara cells can be easily identified by the expression of surfactant protein-C (SP-C) and the Clara cell 10 protein (CC10) respectively [63]–[64]. As expected, our confocal microscopy analysis revealed that all the neoplastic foci were composed mainly by SP-C^+^ cells (Fig. 1). In all cases the SP-C^+^ cells co-expressed the JSRV Env that was localized mainly at the apical surface of the cell (Fig. 1A–C). Despite multiple optical serial section (z stacks images) were analysed for each section, we found that the majority of tumor lesions were formed by cells that did not express CC10 (Fig. 1D). Areas with CC10^+^ cells were detected in 2 of the 6 natural OPA tumours analyzed. However, in both of these cases CC10^+^ positive cells did not show clear expression of the JSRV Env (Fig. 1E–F).

### Age susceptibility to JSRV infection and transformation

Experimentally, OPA can be easily induced in lambs but not in adults [28]–[29], [65]. The incubation period of experimentally induced OPA is directly related to the age of the infected animals [29]. These data can be explained by hypothesizing a differential abundance of the cell targets for viral infection in lambs compared to adult sheep. Alternatively, the target cells for JSRV infection may be present both in lambs and in adults but only in the former, infection is able to progress to neoplastic transformation. In order to begin to address this issue we experimentally infected four newborn lambs and four adult sheep with JSRV and analysed virus-infected cells 10 days post-infection.

**Figure 2:**
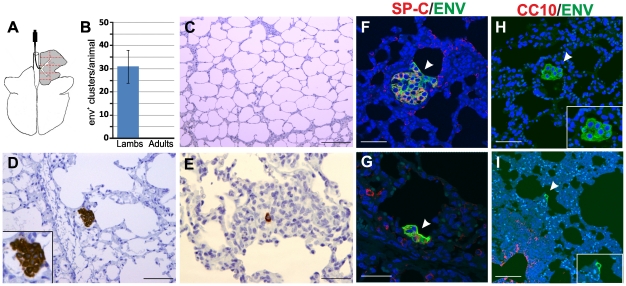
JSRV infection in lambs and adult sheep. (A) Schematic diagram of the sheep lungs. Experimental inoculations were performed administering JSRV by bronchoscopy directly into the accessory bronchus. Tissue samples (1 for each lamb and 2 for each adult sheep) were collected from eight areas (delimited by red lines dotted lines in the panel) of the right cranial lobe. (B) Graph showing the mean number of JSRV Env^+^ clusters per animal as detected by immunohistochemistry in four lambs and four adult sheep (error bars indicate ± SD) 10 days post-infection. (C–E) Immunohistochemistry of JSRV Env^+^ cells in lung sections of adult sheep (C) and lambs (D–E) 10 days post infection. Env expression (characterized by the intra cytoplasmic dark brown colour) was detected in all experimentally infected lambs but not in adult sheep. (F–I) Phenotype of JSRV infected cells in experimentally infected lambs after 10 day post-infection. Panels F–G show lung sections analyzed by confocal microscopy using antibodies towards SP-C (showed in red) and the JSRV Env (showed in green). Nuclei were stained with DAPI and are shown in blue. Arrows indicate JSRV Env+ cells. Panel H–I show lung sections analyzed by confocal microscopy using antibodies towards CC10 (showed in red) and the JSRV Env (showed in green). Inserts show a larger magnification of the area indicated by the arrows. Scale bars in C = 200 µm; D–E = 100 µm; F–G = 47 µm; H = 30 µm; H = 75 µm.

Virus was inoculated directly in the accessory bronchus via bronchoscopy in order to facilitate subsequent detection (Fig. 2A). Animals were euthanized 10 days post-infection and lung samples collected from either 8 (in lambs) or 16 (in adult sheep) regions of the cranial lobe of the lungs to maximise the chances of detecting a small number of virus infected cells and in order to compensate the differences in size between the lambs and adult lungs.

We detected JSRV infected cells by immunohistochemistry using monoclonal antibodies against the viral Env [24], [49]. We were not able to detect any JSRV-infected cells in all the sections derived from the adult sheep used in this experiment (Fig. 2B–C). In contrast, all sections analyzed from each lamb showed JSRV-infected cells (Fig. 2B, D–E). On average, in each lamb we detected 32 clusters of JSRV infected cells ranging in size from 1 to 36 cells (mean 4.9±6.5) with some of them clearly displaying a neoplastic phenotype. Overall these data strongly suggest that the age related susceptibility to OPA is due to the ability of JSRV to infect cells that are much more abundant in the lungs of lambs compared to adult sheep.

We then characterized the phenotype of viral infected cells in the lungs of experimentally infected lambs. We analyzed by immunofluorescence and confocal microscopy lung sections incubated with both antibodies towards SP-C or CC10 and the JSRV Env. In all cases, JSRV Env^+^ cells were also SP-C^+^ (Fig. 2F, G). We were not able to detect any JSRV Env^+^ cell that was also CC10^+^. Some early neoplastic lesions were observed in the respiratory bronchioli but in these cases they were always CC10 negative (Fig. 2H–I). Overall, the data obtained in experimentally infected lambs at the early stages of viral infection are in accordance with the observations made in naturally occurring OPA cases and indicate that cells of the type 2 pneumocytes lineage are infected and transformed by JSRV.

### Age-dependent susceptibility to JSRV infection correlates with the presence of proliferating type 2 pneumocytes

**Figure 3:**
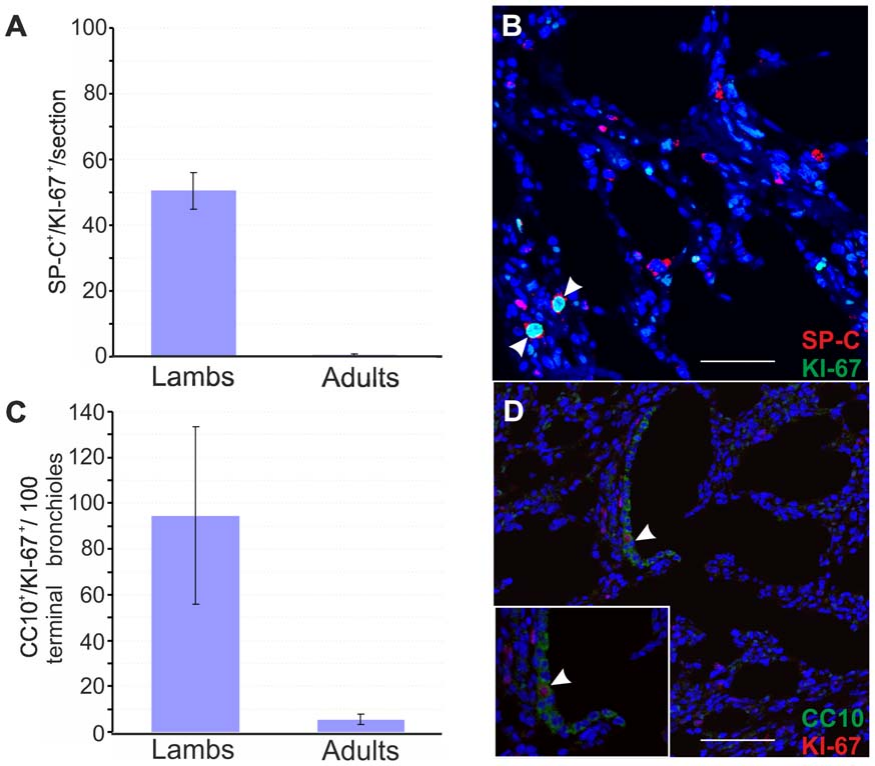
Number of proliferating SP-C^+^ and CC10^+^ cells in healthy lambs and adult sheep. (A) Analysis of proliferating type 2 pneumocytes was performed by counting SP-C/Ki67 double positive cells in 2–4 day-old lambs (n = 4) and adult sheep (n = 2) by confocal microscopy as described in Materials and Methods. 10 sections for each animal were analysed using by confocal microscopy and numbers of double positive cells were normalized to the sectioned area. Results shown are the average numbers of SP-C^+^/Ki67^+^ (± SD) per section for both groups of animals. (B) Representative image of a lung section from a 2 day old lamb analyzed by confocal microscopy using antibodies towards SP-C (showed in red) and Ki67 (showed in green). Nuclei were stained with DAPI and are shown in blue. Note that Ki67 is a nuclear marker and therefore positive signal appears in turquoise in the merged image. Arrows indicate SP-C^+^/Ki67^+^ cells. (C) Analysis of proliferating Clara cells was performed by counting the number of CC10^+^/Ki67^+^ cells in 100 terminal bronchioli per each animal as indicated in Panel A. Results shown are the average numbers of CC-10^+^/Ki67^+^ (± SD) per 100 terminal bronchioli for both groups of animals. (D) Representative image of a lung section from a 2 day old lamb analyzed by confocal microscopy using antibodies towards CC-10 (showed in green) and Ki67 (showed in red). Nuclei were stained with DAPI and are shown in blue. Arrows indicate CC-10^+^/Ki67^+^ cells.

So far, our results showed that lambs are more susceptible to experimentally induced OPA due to the ability of the virus to infect type 2 pneumocytes in lambs but not in adults. Obviously, mature type 2 pneumocytes are present abundantly both in lambs and adults. Therefore, we reasoned that JSRV was able to infect a sub-population of SP-C^+^ that was abundantly present in lambs but not in adult sheep. The normal developed lung is a relatively quiescent organ, with low levels of cell division in the bronchioalveolar epithelium [66]. For a variety of mammals, lungs are not yet mature at birth but continue to develop during a period (“alveolar” stage) where the number of alveoli increases dramatically [67]–[68]. Thus, we hypothesised that JSRV infected lung alveolar proliferating cells instead of post-mitotic type 2 pneumocytes. In order to test this hypothesis, we first analysed by immunofluorescence the mitotic status of type 2 pneumocytes and Clara cells in lambs and adults sheep lungs using antibodies towards the proliferation marker Ki67 [69] in conjunction with either antisera towards SP-C or CC10 (Fig. 3). We found that proliferating type 2 pneumocytes (SP-C^+^/Ki67^+^), addressed here as lung alveolar proliferating cells (LAPCs), were up to 50 times more abundant in newborn lambs compared to adult sheep (p<0.001) (Fig. 3A–B). Also proliferating Clara cell (CC10^+^/Ki67^+^) in the terminal bronchioli were more abundant in lambs compared to adult sheep. We detected 94.5±39 CC10^+^/Ki67^+^ per 100 terminal bronchioli in lambs while there were only 5.5±2.1 CC10^+^/Ki67^+^ per 100 terminal bronchioli in adult sheep (p = 0.004) (Fig. 3C–D).

A subset of SP-C^+^/CC10^+^ putative pulmonary stem cells (known as bronchioalveolar stem cells or BASCs) was identified at the bronchioalveolar junction in mice [43]. We analysed the localization of the proliferating Clara cells in the terminal bronchioli of lambs and sheep and found that they were not localised in a specific area of the terminal bronchioli but randomly distributed. In addition, we could not detect SP-C^+^/CC-10^+^ double-positive cells by confocal microscopy in either lambs or adult sheep, while we were able to identify cells with this phenotype in mice (Fig. S1).

### Lung injury renders adult animals susceptible to JSRV infection and transformation

**Figure 4:**
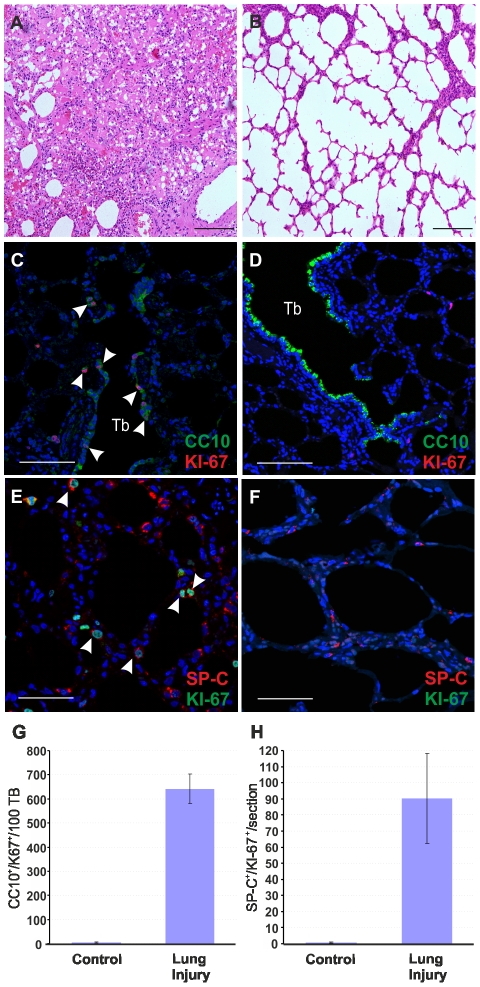
Mild lung injury model in adult sheep. (A–B) Histology of lung sections of sheep treated with 3 methylindole (3MI) (A) and mock-treated controls (B). Treatment with 3MI is characterized by edema and haemorrhagic areas. (C–D) Representative images of lung sections from adult sheep treated with 3MI (C) or mock control (D) analyzed by confocal microscopy using antibodies towards CC10 (showed in green) and Ki67 (showed in red) and the appropriate fluorescent conjugates as described in Materials and Methods. Nuclei were stained with DAPI and are shown in blue. Note in panel C the extensive injury of bronchiolar epithelium (absence of CC10 staining) and proliferation of toxicant resistant Clara cell progenitors (indicated by arrows). Note in both panels C and D the terminal bronchioli (Tb). (E–F) Representative images of lung sections from adult sheep treated with 3MI (E) or mock-treated (F) analyzed by confocal microscopy using antibodies towards SP-C (showed in red) and Ki67 (showed in green). Note in panel E the presence of several SP-C^+^/Ki67^+^ cells. Nuclei were stained with DAPI and are shown in blue. Arrows indicate SP-C^+^/Ki67^+^ cells. (G) Graph representing the quantification of the data analysed by confocal microscopy. Results shown are the average numbers of SP-C^+^/KI-67^+^ (± SD) per section for both groups of animals. (H) Analysis of proliferating Clara cells was performed by counting the number of CC10/Ki67 double-positive cells in 100 terminal bronchioli per each animal. Results shown are the average numbers of CC-10^+^/Ki67^+^ (± SD) per 100 terminal bronchioli for both groups of animals. Scale bars, A–B = 200 µm; E–F = 47 µm; C–D = 89 µm.

So far our data suggested that the presence of LAPCs in lambs is the main factor determining the susceptibility of young animals to JSRV infection as opposed to the resistance observed by adult sheep. Indeed, in the adult lungs, the proliferation rate of the respiratory epithelium is very low [68]. However, the lung has a significant reparative capability and after an injury the LAPC proliferate and play an important role in the tissue regenerative process. We therefore reasoned that we would be able to render adult sheep susceptible to experimental JSRV infection by previous induction of a mild lung injury that would stimulate LAPCs. 3MI is an organ-selective pneumotoxicant that affects specifically type I pneumocytes and bronchiolar epithelial (Clara) cells and it is especially effective in ruminants [69], [70]. Here, to assess the ability of 3MI to induce lung injury and repair we exposed two sheep to this pneumotoxicant and we then assessed lung injury after 48 hours. Histological examination showed diffuse pulmonary edema with scattered hemorrhagic foci (Fig. 4A–B). Next, we assessed the proliferation status of type 2 pneumocytes and Clara cells by verifying co-expression of SP-C or CC10 with the proliferating marker Ki67 by immunofluorescence as described above (Fig. 4C–F). The number of SP-C^+^/Ki67^+^ cells was 90 fold higher in sheep after lung injury as opposed to normal control sheep (p<0.001) (Fig. 4H). The examination of the terminal bronchioli in sheep after 3MI administration revealed that almost 100% of terminal bronchioli contained CC10^+^/Ki67^+^ (Fig. 4G). The total number of CC10^+^/Ki67^+^ cells was more than 100 fold higher in adult sheep after lung injury compared to healthy controls (p = 0.009) (Fig. 4H). Also in adult sheep after lung injury we were not able to identify any SP-C^+^/CC10^+^ double-positive cells (data not shown).

**Figure 5:**
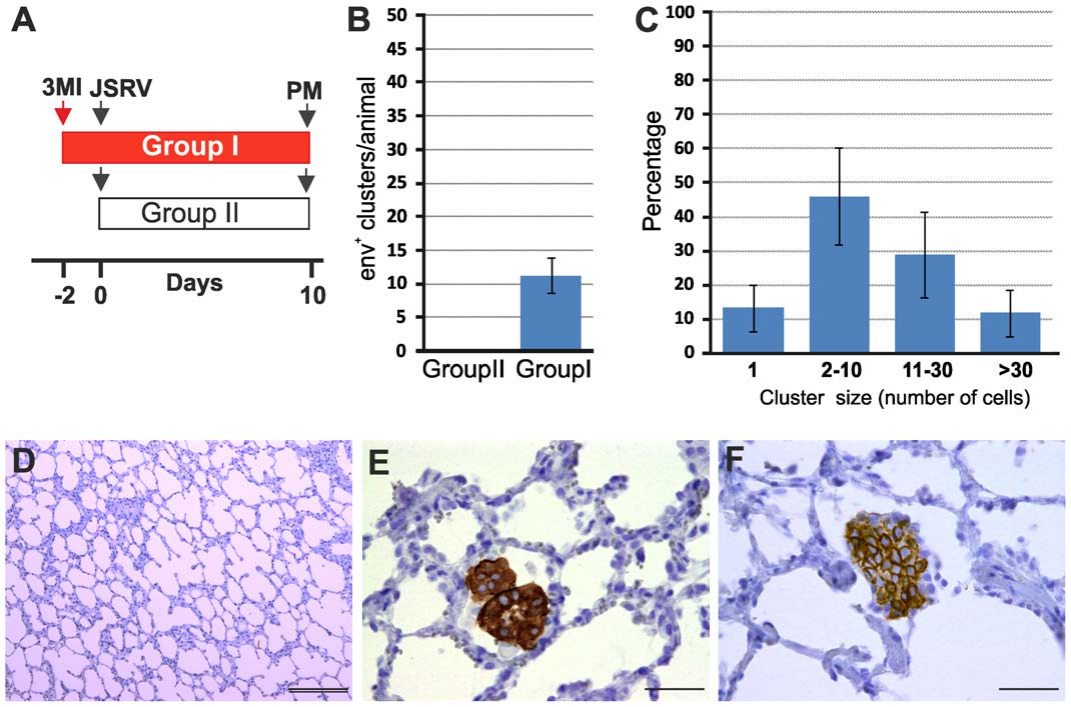
Induction of mild lung injury renders adult sheep susceptible to JSRV infection. (A) Schematic diagram of the study design. Ten adult sheep were randomly allocated in two groups. Sheep in Group I (red bar) were dosed 3MI (red arrow) as described in Materials and Methods while Group II served as control. After 2 days all animals in both groups were infected with JSRV (black arrow) and euthanized 10 days post infection. (B) Graph showing the mean number of JSRV Env^+^ clusters per animal as detected by immunohistochemistry in sheep of Group I and Group II 10 days post-infection (± SD). (C) Graph showing the number of cells forming each cluster of JSRV Env^+^ cells in sheep of Group I. (D–F) Immunohistochemistry of lung sections of adult sheep of group II (D) and group I (E–F, pre-treated with 3MI before JSRV infection) 10 days post-infection as described in Materials and Methods. Env expression (characterized by the intra cytoplasmic dark brown colour) was detected in sheep of Group I but not in sheep of Group II. Scale bars, D = 200 µm; E–F = 100 µm.

Overall, the data presented above indicate that the number of LAPCs, that we identified as target cells of JSRV infection, increase dramatically after mild lung injury. In order to determine whether lung injury may render adult sheep susceptible to JSRV infection, we treated five sheep with 3MI and after 48 h we infected them with JSRV (Group I). Five additional sheep were infected with JSRV without pre-treatment with 3MI (Group II). 10 days after infection animals were euthanized (Fig. 5A). As expected, post-mortem examination revealed no signs of lesions attributed to lung injury. In each animal, the presence of JSRV infection was assessed in 15 sections collected from the cranial lobe by immunohistochemistry. JSRV Env expression was only detected in lung cells of animals that were infected after treatment with 3MI (Fig. 5B, D–F). On average, 10 clusters of JSRV Env^+^ cells (ranging from 1 to 80 cells) were detected in each animal while no JSRV infected cell was detected in those animals that were infected without 3MI pre-treatment (Fig. 5C).

**Figure 6:**
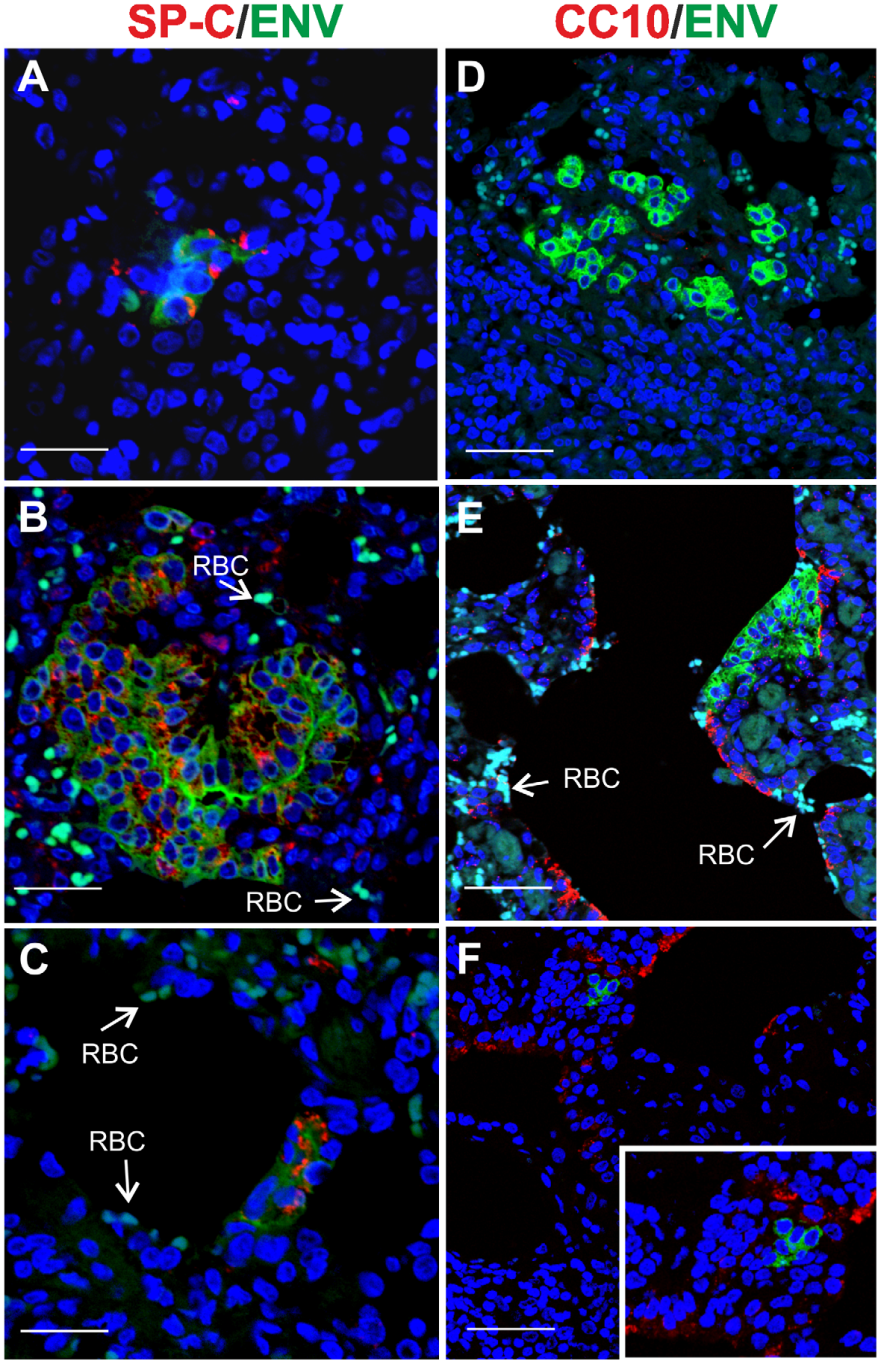
Phenotype of JSRV infected cells in adult sheep with lung injury. Representative images of lung sections from adult sheep pre-treated with 3MI before experimental JSRV infection. (A–C) Sections were analyzed by confocal microscopy using antibodies towards SP-C (showed in red) and the JSRV Env (showed in green). Nuclei were stained with DAPI and are shown in blue. Arrows indicate autofluorescent red blood cells (RBC). (D–F) Sections analyzed as above using antibodies towards CC10 (showed in red) and the JSRV Env (showed in green). Nuclei were stained with DAPI and are shown in blue. Scale bars, A–B = 47 µm; C = 89 µm; D = 26 µm; E = 33 µm and F = 25 µm.

By immunofluorescence and confocal microscopy we found that all JSRV infected cells were SP-C positive (Fig. 6A–C). None of the JSRV Env^+^ cells were CC10^+^ (Fig. 6D–F), despite the high number of proliferating Clara cells induced by 3MI and the presence of numerous infected cells localized in the terminal bronchioli.

### JSRV preferentially infects dividing cells *in vitro*

**Figure 7:**
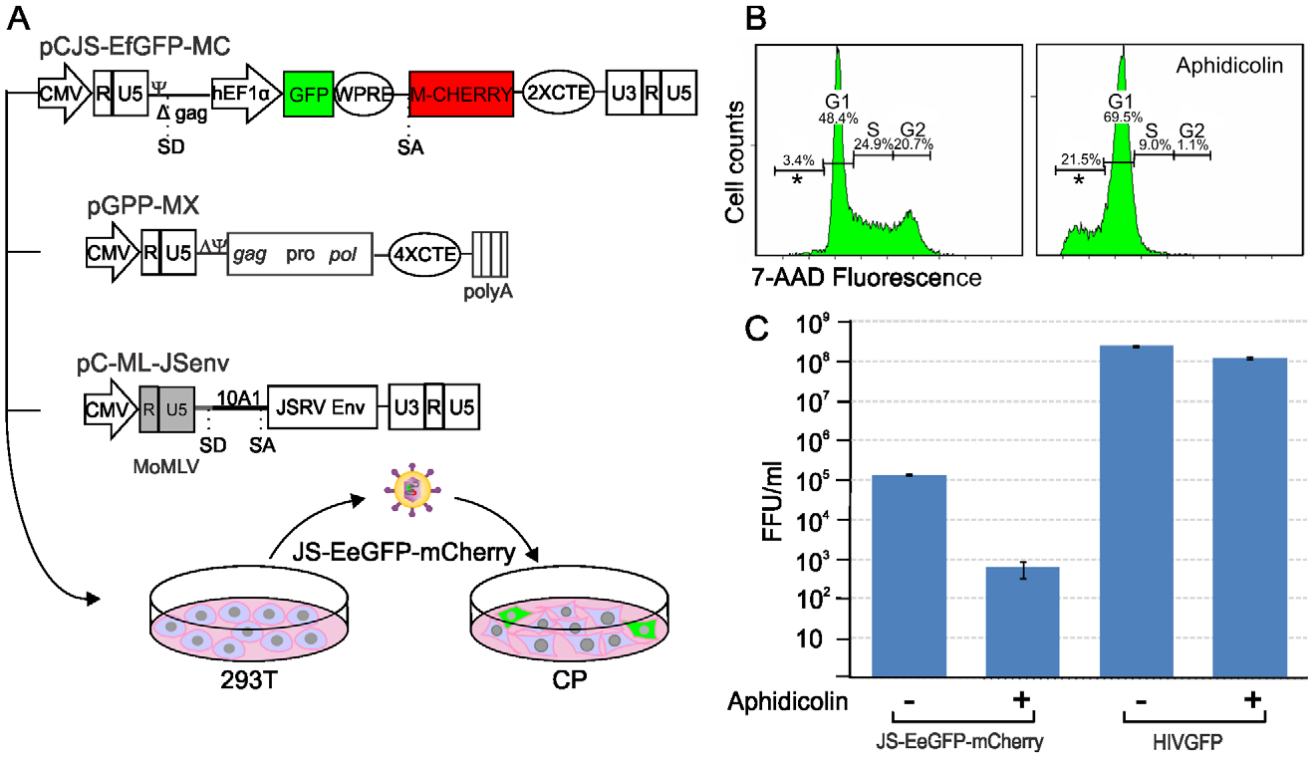
Infectivity of JSRV in proliferating and mitotic arrested cell. (A) Schematic representation of the experimental design. A JSRV-based vector was derived by transfecting 293T with pJS-EFGFP-MC, pGPP-MX-4CTE and pC-MLV-JSenv as described in the materials and methods. The resulting vector, JS-eGFP, was then used to infect synchronized choroid plexus cells (CP) cultures in the presence or absence of aphidicolin. (B) Histograms showing the DNA content of CP cells with or without Aphidicolin (5 µg/ml for 24 hours). The DNA content was measured by 7AAD staining and flow-cytometry analysis and provides an indication of the cell cycle. The x and y axis represent the relative DNA content and the cell counts. (C) Graph showing the transduction efficiency (expressed as fluorescence forming foci/ml) of JS-eGFP in proliferating or mitotic arrested CP as described in Materials and Methods.

Our data have shown that JSRV infects LAPCs but not the overwhelming majority of type 2 pneumocytes which divide very slowly. These data could be explained mechanistically by the fact that the majority of retroviruses, with the exception of lentiviruses [71], infect more efficiently cells that are in mitosis [72]–[73]. The proliferation rate of type 2 pneumocytes is very low in adults under normal conditions. On the other hand the higher proliferative rate of LAPCs during post-natal development or tissue repair in the adult would facilitate JSRV infection. Experiments with JSRV *in vitro* are hindered by the lack of a convenient tissue culture system for the propagation of this virus [32]. Therefore, we constructed a convenient JSRV-derived viral vector (JS-EeGFP-mCherry) in order to easily quantify JSRV infection in proliferating and non-proliferating cells. JS-eGFP-mCherry was derived by transiently transfecting 293T cells with (i) a packaging plasmid (pGPP-MX-4CTE) devoid of the JSRV packaging signal (Ψ) and expressing the viral Gag, Pro and Pol; (ii) a plasmid providing the JSRV Env *in trans* (pC-ML-JSenv, also devoided of Ψ), and (iii) the packaged JSRV vector (pCJS-EFGFP-MC) that upon infection and integration expresses eGFP under the control of an internal promoter (Fig. 7A). JS-eGFP-mCherry viral particles were then used to infect synchronized SCP cells in the presence or absence of a drug that, at the concentration used in this study, arrests cells in the G1 phase (aphidicolin) (Fig. 7B). Consistently, JS-eGFP-mCherry was able to transduce actively dividing SCP cells approximately 200 times more efficiently (p = 0.002) than the same cells where mitosis was arrested with aphidicolin while only minor differences between treated and untreated cells were observed with the lentivirus vector HIV-GFP (Fig. 7C).

## Discussion

In this study we have investigated the pathogenesis of a unique virus-induced lung adenocarcinoma and obtained data that have a broad significance in pulmonary biology, carcinogenesis and retroviral pathogenesis. Most adenocarcinomas in humans display cells expressing type 2 pneumocytes or Clara cell markers but it is not completely clear whether the neoplasm arises from a stem cell that is able to differentiate into both cell types, or from a committed progenitor or from the fully differentiated cell compartments [74]. In this study, we identified the target cells of JSRV infection and transformation *in vivo* as proliferating cells of the type 2 pneumocytes lineage (SP-C^+^/Ki67^+^, LAPC). In addition, we showed that the age-related susceptibility to experimental OPA induction is directly related to the abundance of LAPCs. Importantly, induction of mild injury to the respiratory epithelium increased dramatically the number of LAPCs in adult sheep and rendered these animals susceptible to JSRV infection and transformation. We have not found evidence that CC10^+^/Ki67^+^ cells are infected and transformed by JSRV. Furthermore, we found that the CC10^+^ cells that are found in a proportion of late stages OPA tumours are not expressing JSRV proteins and may therefore not be true tumour cells, at least in the cases we examined.

Our data provide important consideration for pulmonary biology and carcinogenesis. We infer from our study that at least in sheep, type 2 pneumocytes and Clara cells have two distinct populations of proliferating progenitor cells committed to the alveolar and the bronchiolar lineages. From this study, we cannot determine whether the LAPCs are progenitor committed solely to type 2 or type 1 pneumocytes. We showed that lung adenocarcinoma can originate from an alveolar proliferating cell of the alveolar lineage, rather than from a bronchioalveolar stem cell postulated to originate both type 2 pneumocytes and Clara cells. Studies in mice have identified a population of putative stem cells that are both SP-C^+^ and CC10^+^ (bronchioalveolar stem cells, BASCs) located at the bronchioalveolar duct junction [43]. Based on *in vitro* analysis, BASCs were hypothesised to give rise to Clara cells, alveolar type 2 cells and be the cell originating lung adenocarcinoma [43]. On the other hand, studies using genetic lineage-labelling experiments in mice, supported a model where bronchioli and alveoli are maintained and repaired distinctively by Clara cells and LAPCs respectively [44], [75]. The presence of BASCs in humans has not been confirmed and in general the biological relevance of BASCs is object of debate [44]–[45]. In our study, by confocal microscopy, we have not been able to detect SP-C^+^/CC10^+^ in sheep while we were able to detect cells with this phenotype in mice (Fig. S1). We cannot rule out the presence of a rare bronchioalveolar stem cell (SP-C^+^/CC10^+^) able to differentiate in both type 2 pneumocytes and Clara cell progenitors in sheep. We also cannot rule out the presence in sheep of phenotypically uncharacterized pulmonary stem cells. However, if these cells exist in the sheep, they are very rare and unlike LAPCs they do not appear to play a major role in OPA. Interestingly, from the anatomical and histological point of view the human lungs are more comparable to the sheep lungs as opposed to the mice lungs [76]–[77].

We showed with experiments *in vitro* that JSRV, similarly to other retroviruses, infects preferentially cells in active mitosis. These experiments provide a mechanistic explanation to the observation that JSRV infects readily LAPCs but not mature type 2 pneumocytes.

As mentioned before, JSRV is a unique oncogenic virus as it possesses the viral Env (a structural protein) that behaves as a functional dominant oncoprotein both *in vitro* and *in vivo*. In general, viral oncoproteins are non structural proteins whose expression is not linked to productive infection. It would be detrimental from an evolutionary point of view of the virus, to have productive viral infection and carcinogenesis as strictly mutually dependent events (viral replication would in this case lead to the death of the infected host). Onset of lung adenocarcinoma in JSRV-infected animals could therefore be viewed as either “accidental” (similarly to other retrovirus-induced tumors) or “essential” in order to allow virus spread among susceptible hosts. Although these two alternative hypotheses are not necessarily mutually exclusive, the data obtained in this study and accumulated over the years on JSRV/OPA, strongly suggest that tumor induction plays an important part in the evolutionary strategies used by the virus to persist in the sheep population. In previous studies we have shown that development of OPA in the field occurs only in a minority of the JSRV-infected sheep [39]. On the other hand, animals with OPA produce lung secretions containing abundant amounts of infectious JSRV particles that pour freely from the nostrils of the affected sheep [41], [78]–[79]. The data from this paper strongly suggests that clinical OPA develops in natural conditions as a result of viral infection only when LAPCs are available to the virus: in young lambs during post-natal development or in the presence of an injury to the bronchioalveolar epithelium. Importantly, as mentioned in the introduction, JSRV proteins are detected readily only in the tumour cells of OPA affected animals (and in the LAPCs as shown in this study) [38] although low levels of virus infection and protein expression are detectable in cells of the lymphoreticular system of animals with or without clinical OPA. We and others have shown that the JSRV LTRs are the main determinants regulating the tight cell-specific expression pattern displayed by this virus. The JSRV LTRs contains lung-specific enhancer binding motifs that are preferentially active in cell lines derived from transformed type 2 pneumocytes [80]–[83]. In addition, in transgenic mice, reporter gene expression driven by the JSRV LTR has been detected specifically in type 2 pneumocytes [84]. Thus, JSRV-host equilibrium has been reached by a combination of factors. JSRV has evolved a structural protein that is a powerful oncoprotein but only when expressed at high levels in the LAPCs, which are relatively rare cells in the adult healthy sheep. Therefore, JSRV has a limited window of opportunity to infect the target cells of the host that allow high level of viral expression (and that can be consequently transformed). At the same time, onset of lung adenocarcinoma in a minority of the infected animals allows an amplification of the cells that can produce infectious virus and therefore it is a likely evolutionary mechanism that helps JSRV to persist in the population.

It is important to note that in natural conditions, sheep with OPA present consistently a variety of other parasitic, bacterial or viral infections [9]. Classically, these infections were considered as “secondary” to JSRV infection. We suggest instead that in the adult, the induction of an injury to the respiratory epithelium by various pathogens substantially increases the number of LAPCs and renders adult sheep susceptible to JSRV-induced transformation, similarly to what we have shown experimentally in this study with the pneumotoxicant 3MI. Thus, inflammation induced by different pathogens is the “primary” event for OPA induction. It is feasible that in animals already infected with JSRV the virus present in lymphoreticular cells is able to spread to injured tissues where it can infect and transform alveolar progenitor cells actively involved in repairing the epithelium.

In conclusion, this work provided unique insights into pulmonary physiology, lung cancer, and retrovirus pathogenesis and is another telling example where viruses have helped us to understand fundamental aspects of host biology.

## Supporting Information

Figure S1 Detection of SP-C and CC10 in cells of the bronchioalveolar duct junctions of sheep and mice lungs. A. Representative images of lung sections from adult mice. Sections were analyzed by confocal microscopy using antibodies towards CC-10 (green) and SP-C (red). CC10^+^/SPC^+^ double positive cells bronchioalveolar stem cells (BASCs) are located at the bronchioalveolar duct junction (BADJ). Arrows point to BASC cells. Nuclei were stained with DAPI and are shown in blue. Scale bar, 75 µm. B. Representative image of lung sections from adult sheep. Sections were analyzed by confocal microscopy using antibodies towards CC-10 (green) and SP-C (red). Nuclei were stained with DAPI and are shown in blue. No SP-C/CC10 double-positive cells are detectable. Scale bar, 62 µm.
(TIF)
