# Supplementary figures and images for: Lung Adenocarcinoma Originates from Retrovirus Infection of Proliferating Type 2 Pneumocytes during Pulmonary Post-Natal Development or Tissue Repair
Source: PLoS Pathog. 2011 Mar 31;7(3):e1002014. doi: 10.1371/journal.ppat.1002014 (PMC3068994; doi:10.1371/journal.ppat.1002014)

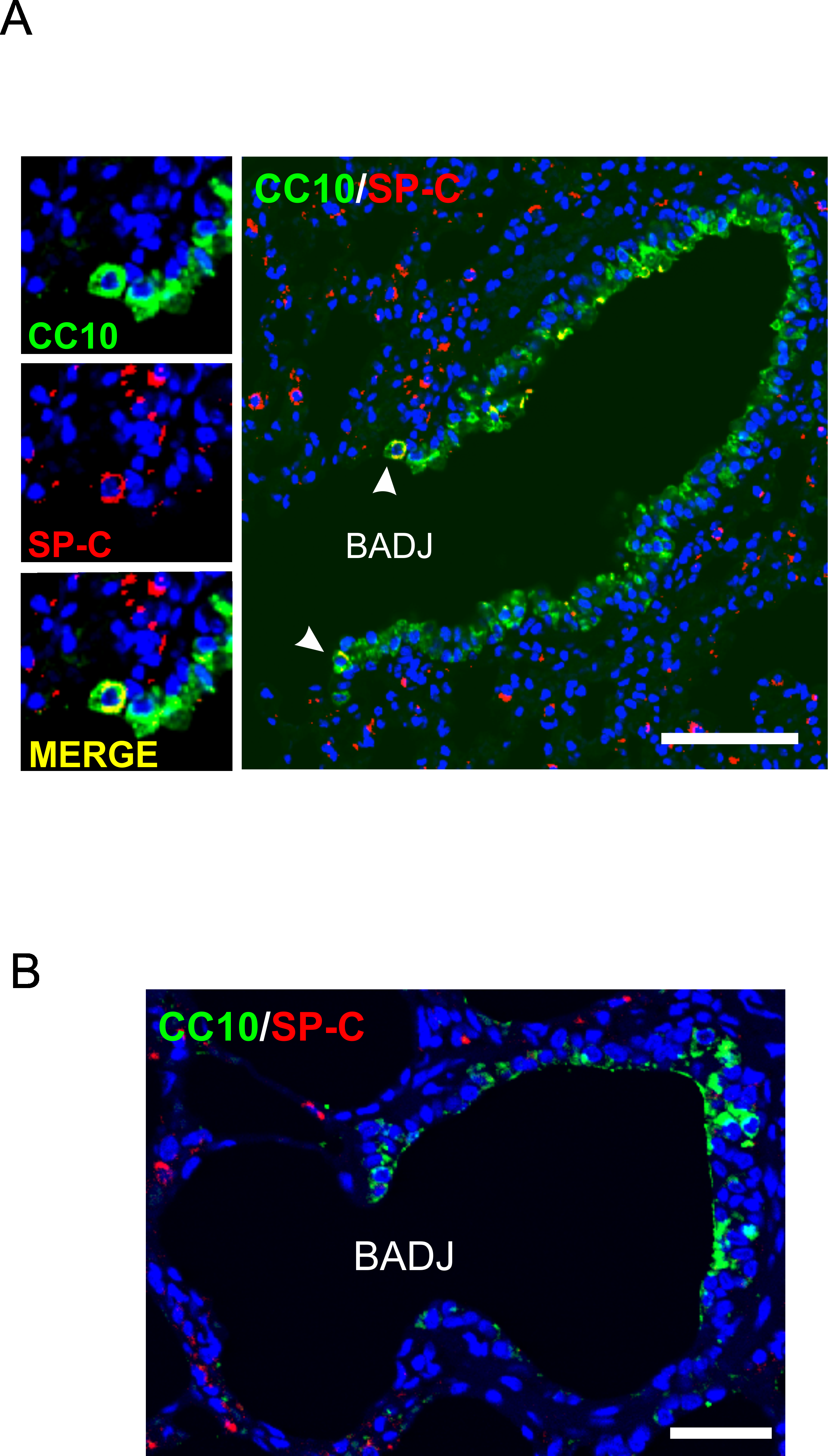

Supplement: Figure S1 — Detection of SP-C and CC10 in cells of the bronchioalveolar duct junctions of sheep and mice lungs. A. Representative images of lung sections from adult mice. Sections were analyzed by confocal microscopy using antibodies towards CC-10 (green) and SP-C (red). CC10+/SPC+ double positive cells bronchioalveolar stem cells (BASCs) are located at the bronchioalveolar duct junction (BADJ). Arrows point to BASC cells. Nuclei were stained with DAPI and are shown in blue. Scale bar, 75 µm. B. Representative image of lung sections from adult sheep. Sections were analyzed by confocal microscopy using antibodies towards CC-10 (green) and SP-C (red). Nuclei were stained with DAPI and are shown in blue. No SP-C/CC10 double-positive cells are detectable. Scale bar, 62 µm. (TIF) [file ppat.1002014.s001.tif]
